# Identifying the Threshold of Iron Deficiency in the Central Nervous System of the Rat by the Auditory Brainstem Response

**DOI:** 10.1177/1759091415569911

**Published:** 2015-02-19

**Authors:** Allison R. Greminger, Margot Mayer-Pröschel

**Affiliations:** 1 Department of Environmental Medicine, University of Rochester, Rochester, NY, USA; 2 Department of Biomedical Genetics, University of Rochester, Rochester, NY, USA

**Keywords:** iron deficiency threshold, iron-deficient diet, auditory brainstem response, auditory nerve, ferritin

## Abstract

The deleterious effects of anemia on auditory nerve (AN) development have been well investigated; however, we have previously reported that significant functional consequences in the auditory brainstem response (ABR) can also occur as a consequence of marginal iron deficiency (ID). As the ABR has widespread clinical use, we evaluated the ability of this electrophysiological method to characterize the threshold of tissue ID in rats by examining the relationship between markers of tissue ID and severity of ABR latency defects. To generate various levels of ID, female Long-Evans rats were exposed to diets containing sufficient, borderline, or deficient iron (Fe) concentrations throughout gestation and offspring lifetime. We measured hematological indices of whole body iron stores in dams and offspring to assess the degree of ID. Progression of AN ID in the offspring was measured as ferritin protein levels at different times during postnatal development to complement ABR functional measurements. The severity of ABR deficits correlated with the level of Fe restriction in each diet. The sufficient Fe diet did not induce AN ID and consequently did not show an impaired ABR latency response. The borderline Fe diet, which depleted AN Fe stores but did not cause systemic anemia resulted in significantly increased ABR latency isolated to Peak I.The low Fe diet, which induced anemia and growth retardation, significantly increased ABR latencies of Peaks I to IV. Our findings indicate that changes in the ABR could be related to various degrees of ID experienced throughout development.

## Introduction

Iron deficiency (ID) is the most common micronutrient deficiency in the world and is especially prevalent during early brain development, particularly as daily iron requirements triple by the third trimester (World Health Organization, 2001). Iron serves many functions in the body both systemically, as well as on a tissue specific level. In the central nervous system (CNS), iron is an essential cofactor for many enzymes including those involved in energy metabolism, neurotransmitter synthesis, and the process of myelination (Conrad et al., 2000; Rao et al., 2003; Eden, 2005; Brotanek et al., 2007; Tran et al., 2008; Wu et al., 2008; Collard, 2009; Todorich et al., 2009; Tran et al., 2009; Kumari et al., 2012; Tran et al., 2012; Callahan et al., 2013; Polin et al., 2013; Radlowski and Johnson, 2013; Rao et al., 2013). Therefore, the impact of ID during vulnerable periods of neurodevelopment can be significant, and early gestational ID has been associated with abnormalities in neuronal development in both children and animals (Felt and Lozoff, 1996; Lozoff, 2000; Algarin et al., 2003; Walter, 2003; Lozoff and Georgieff, 2006; Rosato-Siri et al., 2010; Mihaila et al., 2011; Lee et al., 2012; Amin et al., 2013; Pisansky et al., 2013; Greminger et al., 2014; Jougleux et al., 2014)

Similar to other nutritional deficiencies, ID occurs as a spectrum beginning with tissue iron store depletion and, if unsupplemented, progressing to impaired erythropoiesis or anemia (Connor et al., 2001; Collard, 2009; Ganz, 2013). All stages of maternal ID have been associated with adverse effects on the offspring, but only the most severe stage of ID can be reliably diagnosed via maternal blood or cord blood samples (Scholl and Reilly, 2000; Gambling et al., 2003; Lisle et al., 2003; Gambling et al., 2004; Andersen et al., 2006; Felt et al., 2006; Zhou et al., 2006; Zimmermann et al., 2007; Banhidy et al., 2010). Marginal maternal iron intake leads to low fetal Fe levels in CNS tissue but is below the threshold of generating anemia. This stage of marginal ID is clinically difficult to diagnose and remains for the most part undetected. This is of great concern as infants born with suboptimal iron stores may be at a greater risk of impaired neural maturation. Therefore, there is a paramount need for diagnostic neurofunctional screening that could identify low CNS iron stores, despite normal systemic clinical endpoints.

Global behavioral tests often shed little information on the specific abnormalities produced by developmental insults. The auditory brainstem response (ABR) is a noninvasive technique that provides objective measurement of auditory thresholds and neuronal transmission speed along the auditory pathway from the end-organ to the brainstem and higher auditory cortex (Jougleux et al., 2011). Signal processing begins in the cochlea, which functionality can be determined by the distortion-product otoacoustic emissions (DPOAEs) amplitudes. Changes in DPOAEs indicate damage to outer or inner hair cells, which are responsible for loss of auditory thresholds. In the absence of haircell damage, signal processing and conduction velocity are mainly dependent on axon diameter, myelination, and synapse integrity of the auditory nerve (AN; Lasky et al., 1995; Cankaya et al., 2003). In contrast to techniques that measure overall developmental delays, which could be the result of multiple factors, the ABR is advantageous because it provides localized functional measures at multiple synapse levels along the CNS pathway (Moore, 1987; Angulo-Kinzler et al., 2002). The series of nuclei involved in AN transmission are represented by the number and amplitude of individual wave components that vary by age and species (Jerger and Hall, 1980; Corwin et al., 1982; Moore, 1987; Jiang et al., 1991). Peak I (PI) represents the activity of the AN nerve, whereas Peaks III to V are associated with higher auditory CNS centers such as the inferior colliculi (PIII) and auditory brainstem (PV) (Moore, 1987). Absolute latency of these waves or peaks can be measured, as well as the interpeak intervals, which are used to calculate the speed of transmission (Warren, 1989).

While the ABR is employed as a diagnostic tool for speech and language learning deficits (Coenraad et al., 2011; Li et al., 2011; McCandless, 2011; Marcoux, 2011), its value to determine impairments associated with ID in humans remains unclear. Some studies have found significant changes in the ABR response in ID infants (Roncagliolo et al., 1998, 2000; Algarin et al., 2003; Cankaya et al., 2003; Jorgenson et al., 2005; Lozoff et al., 2006; Amin and Guillet, 2007; Amin et al., 2010; Madan et al., 2011), while others found no such correlation (Sarici et al., 2001; Kurekci et al., 2006; Berglund et al., 2011). The discrepancies are likely due to differences in severity or duration of ID in the cohorts examined. Support for this comes from various animal studies where we and others have found that both severe as well as marginal ID affect the ABR response and that the impairment was greater when the ID occurs at the onset of pregnancy and thus affects the early states of gestation (Jougleux et al., 2011; Mihaila et al., 2011; Lee et al., 2012). As these studies employed different animal strains and different feeding paradigms that lead to various degree of ID, it remains difficult to draw firm conclusions about the degree of ID and the resulting impact on the ABR.

The goals of this study presented here were twofold: (a) we wanted to determine the threshold of ID during gestation and postnatal development that would be severe enough to induce CNS tissue ID but would not result in systemic anemia in the offspring, and (b) we aimed to evaluate whether the various degrees of ID established in the offspring would have a corresponding impact on their ABR profiles.

To achieve this, we employed an animal model in which we could keep the time of exposure constant but were able to manipulate the degree of ID. We found that functional responses to the variable iron diets correlated well with both the absolute concentration of iron in the diet and the molecular indicators of tissue ID. We also found that ABR profiling was a sensitive tool to detect possible brain maturation impairments in the offspring even if a clinical state of ID anemia was not indicated.

## Materials and Methods

### Animal Handling and Diets

Female Long-Evans rats (8 weeks) from Charles River Laboratories, (Wilmington, MA) were pair housed in plastic filter-top cages on corn-cob bedding (Pur-o’Cel, Maumee, OH) under humidity and temperature controlled conditions (12 hr light/dark cycle). Animals were housed in a facility accredited by the Association for Assessment and Accreditation of Laboratory Animal Care with all protocols approved by the University Committee on Animal Resources at the University of Rochester. For enrichment, females and their offspring were given cotton square nestlets with every cage change throughout the study. Starting 2 weeks prior to breeding and continuing throughout gestation and lactation, females were fed ad libitum an iron-sufficient (IS) diet containing 240 µg Fe/g diet or one of three iron-deficient diets (IDDs) containing 6, 20, and 30 µg Fe/g, respectively (Harlan Laboratories, see Supplemental Material Table S1). At 11 weeks of age, females were bred to age-matched Long-Evans males, and the presence of vaginal plugs was considered indicative of pregnancy and considered gestational day (GD) 1. Weight was recorded on GD1 and measured as a percentage of GD1 weight gain at GD7, 14, and 18.

At delivery, litter size was recorded and offspring were weighed at postnatal day (P) P7, 14, 21, and 40. Pups were weaned at P21 and provided lifetime ad libitum access to the same respective diet as their dam until sacrifice. Offspring of dams fed the IS diet or ID diet (6, 20, or 30 µg Fe/g) are designated as the IS group (total of 8 litters) or IDD group (total of 30 µg Fe/g = 4, 20 µg Fe/g = 4, and 6 µg Fe/g = 3 litters), respectively. Analyses were performed on offspring of mixed sex, with equal numbers of each sex used per endpoint, where possible.

### Hematological Analysis

Dams at GD15 were lightly anesthetized with isofluorane and restrained while blood (∼100 µL) was extracted from the jugular vein into EDTA coated tubes. Offspring were asphyxiated with carbon dioxide (CO_2_), and whole trunk blood was collected after decapitation at P14, 21, and 40 into potassium-EDTA coated microtubes. Hematocrit (HCT) and hemoglobin (HGB) were analyzed according to the manufacture’s guidelines (Heska®, Loveland, CO) with experimental controls used for reference values.

### Western Blot Analysis

For tissue harvest, animals were asphyxiated with CO_2_, at P14, 21, and 40, rapidly decapitated, and the cochlea separated from the tympanic bulla and otic capsule. Both cochlea from each animal were combined and mechanically lysed in two volumes of XDP lysis buffer and one volume of 0.5 mm glass beads (Next Advance, Averill Park, NY). Lysates were centrifuged, and the protein, which represents at that stage mainly the AN, was separated and stored at −80℃ until analyses. Protein concentration was determined using the DC™ Protein Assay Kit (Bio-Rad, Hercules, CA), and samples (35 µg) were resolved on 4% to 12% bis-tris NuPage gels (Invitrogen, Grand Island, NY). Membranes were probed for anti-ferritin heavy chain (FTH; 1:1000 dilution) for 16 hr at 4℃ (Cell Signaling Technology #3998, Danvers, MA). Immunoreactive proteins were quantified by densitometry using ImageJ software (NIH, Bethesda, MD). Samples were normalized to β-Actin as a loading control and displayed as a ratio to age-matched IS control tissue. Different tissue samples were used for each diet group for each replicate Western blot comparison.

### Auditory Brainstem Response

Testing of the ABR was performed on P40 offspring of each diet. This age was chosen as our previous rodent models of moderate ID showed a significant decrease in neuronal conduction velocity specifically at this age (Lee et al., 2012). Offspring were lightly sedated using a ketamine (6 mg/kg BW)/xylazine (0.5 mg/kg BW) mix for a period of ∼1 hr. Thermal regulation of body temperature was achieved using a heating pad during testing sessions. ABR waveforms were evoked with trapezoidal wave pure tone pips at 30 cycles per second, delivered at frequencies of 8, 16, and 32 kHz (optimal hearing range in rats; Heffner and Heffner, 2007) at 70 decibels (dB SPL), through high-frequency transducers at a distance of 5 cm at a perpendicular angle to the left pinna. Subdermal needle electrodes (Platinum 10 mm, 30 gauge monopolar; Grass Technologies, Middleton, WI) were inserted at the cranial vertex, and in the muscle posterior to the left pinna, with a ground electrode placed in the gluteus maximus muscle. Responses from the left ear were amplified (10,000×), filtered (100 Hz–3 kHz), and averaged using the SmartEP data-acquisition software (Intelligent Hearing Systems, Miami, FL). In total, 1,024 responses were averaged (with alternating stimulus polarity) using an “artifact reject” that discards responses when peak-to-peak amplitude exceeds 15 μV as previously described (Jones et al., 2006). Left ear-only recordings were chosen for this study, as multiple previous experiments showed no evidence of unilateral effects due to ID on ABR responses (unpublished observation).

### Statistical Analyses

Data were appropriately transformed if analysis indicated significant deviation from normality or equal variance. Statistical analysis of endpoints was done using one-way (within age or diet) or two-way analysis of variance (Age × Diet or Diet × Frequency) where appropriate and, in the presence of significant main effects or interactions (*p* < .05), post hoc tests (Holm-Sidak) were used to determine the nature of the effect. Multiple comparisons between diets in post hoc tests were limited to comparison to IS controls within age or frequency.

## Results

### Dietary Iron Availability Only Mildly Affects the Pregnant Dam

We found no significant effect of any diet on gestational weight gain or HGB concentrations 1 week before delivery (Table 1). There were no significant differences in the amount of diet consumed, and administration of the purified diets resulted in a gestational average daily intake of 4.4, 0.498, 0.350, and 0.113 mg Fe for the 240, 30, 20, and 6 µg Fe/g diets, respectively. However, the 6 µg Fe/g IDD significantly reduced HCT by approximately 14% in the IDD-6 dams relative to all the other groups (*p* < .05). The pregnancy outcome was not affected by any of the diets with respect to litter size, gestational length, or gender distribution.

**Table 1.** Maternal and Birth Outcomes in Dams Fed the IS or ID Diets.

	Diet							
	N	IS–240	N	IDD–30	N	IDD–20	N	IDD–6
Diet Consumed, g/day	4	18.5 ± 2.1	4	16.6 ± 1.7	4	17.5 ± 1.9	3	18.8 ± 1.4
Dam weight gain, %								
GD2–GD7	8	13.2 ± 0.5	4	9.2 ± 2.4	4	12.7 ± 1.7	3	8.4 ± 0.4
GD7–GD14	8	19.2 ± 0.8	4	17.3 ± 1.6	4	23.0 ± 1.6	3	14.9 ± 1.2
GD14–GD21	8	32.4 ± 0.8	4	24.6 ± 2.3	4	34.5 ± 2.6	3	28.7 ± 1.0
GD15 HCT, %	4	38.5 ± 1.1	4	37.2 ± 1.6	4	38.1 ± 0.6	3	33.2 ± 2.3*^#^*
GD15 HGB, g/dL	4	14.3 ± 0.6	4	12.7 ± 1.7	4	14.2 ± 0.2	3	12.9 ± 0.3
Gestation length, *d*	8	22.4 ± 0.3	4	22.7 ± 0.3	4	23.3 ± 0.3	3	23.5 ± 0.5
Litter size, *n*	8	13.3 ± 0.4	4	11.6 ± 1.7	4	11.7 ± 1.2	3	13.0 ± 1.1
Gender distribution, *n*								
Male	8	6.9 ± 0.8	4	6.0 ± 2.1	4	6.2 ± 1.2	3	8.5 ± 0.5
Female	8	6.4 ± 0.5	4	5.3 ± 0.7	4	4.4 ± 1.1	3	5.3 ± 0.3
Postnatal mortality^a^	8	0.67 ± 0.67	4	0.83 ± 0.57	4	0.33 ± 0.33	3	6.00 ± 2.18*^#^*

*Note*. Results are given as mean ± SEM. N represents number of dams per diet group. ^#^*p*<.05 vs. all groups, Holm-Sidak. IS = iron sufficient; IDD = iron-deficient diet.

^a^Average number of deaths per litter.

There was, however, a significant difference in offspring postnatal mortality, the 6 µg Fe/g IDD compared with all other groups (*p* < .05), with almost 50% of offspring expiring before the conclusion of the study at P40.

### Weight Gain and Hematological Outcomes in the Offspring Are More Sensitive to Changes in Dietary Iron Concentrations Than in Dams

**Figure 1.**
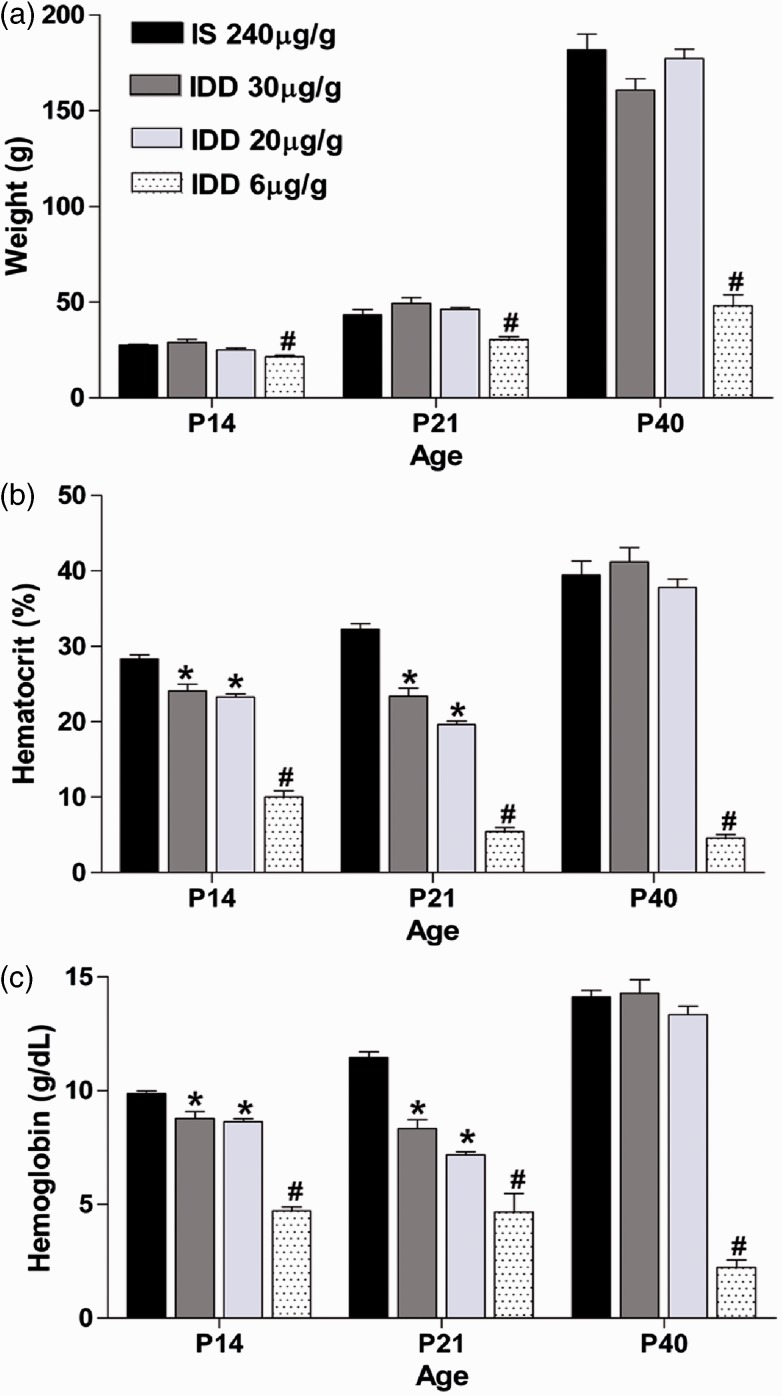
Postnatal growth and hematological parameters in offspring fed an IDD. (a) Weight of offspring at P14, 21, and 40. Data are mean ± *SEM* for *n* = IS: 12–17, IDD-30: 10–11, IDD-20: 11–18, and IDD-6: 4–8 rats per group, per age. Hematocrit (b) and hemoglobin (c) values in offspring at P14, 21, and 40. *p < .05 versus age-matched 240-IS group, ^#^*p* < .05 versus all other diet groups within each age. Data are mean ± *SEM* for *n* = IS: 8–21, IDD-30: 6–9, IDD-20: 10–11, and IDD-6: 3–7 rats per group, per age. IS = iron sufficient; IDD = iron-deficient diet.

Intake of a diet containing 240, 30, or 20 µg Fe/g during pregnancy, weaning, and early postnatal growth leads to a nearly 10-fold increase in body weight from P14 to P21 (main effect of age *p* < .001). We found a significant diet by age interaction (*p* < .001), indicating that the impact of age on postnatal weight is dependent on the diet group. In contrast, if the diet contained only 6 µg Fe/g, the offspring showed significant growth retardation that worsened with age. Surviving pups showed 20% to 70% reduction in body weight relative to the IS control group from P14 to P40, respectively (Figure 1(a)).

Both HCT and HGB values significantly increased with age in the 240 µg Fe/g control diet (main effect of age *p* < .001, diet by age interaction *p* < .001). We did observe a treatment-related effect on HCT and HGB levels at P14 and P21 with respect to the intermediate diets (30 and 20 µg Fe/g diet; Figure 1(b) and (c)). However, these decreases were both mild and transient as they completely recovered by P40, relative to IS offspring. This compensation occurred despite remaining on the same respective deficient diets during this time.

In line with the dramatic lack of weight gain and increased offspring mortality, the 6 µg Fe/g IDD displayed progressive decreases in both HCT and HGB values by 60% to 80% relative to IS offspring from P14 to P40, indicating severe anemia (Figure 1(b) and (c)).

### Tissue Ferritin Levels Correlate Well but Not Precisely With Hematological Iron Availability Indicators

**Figure 2.**
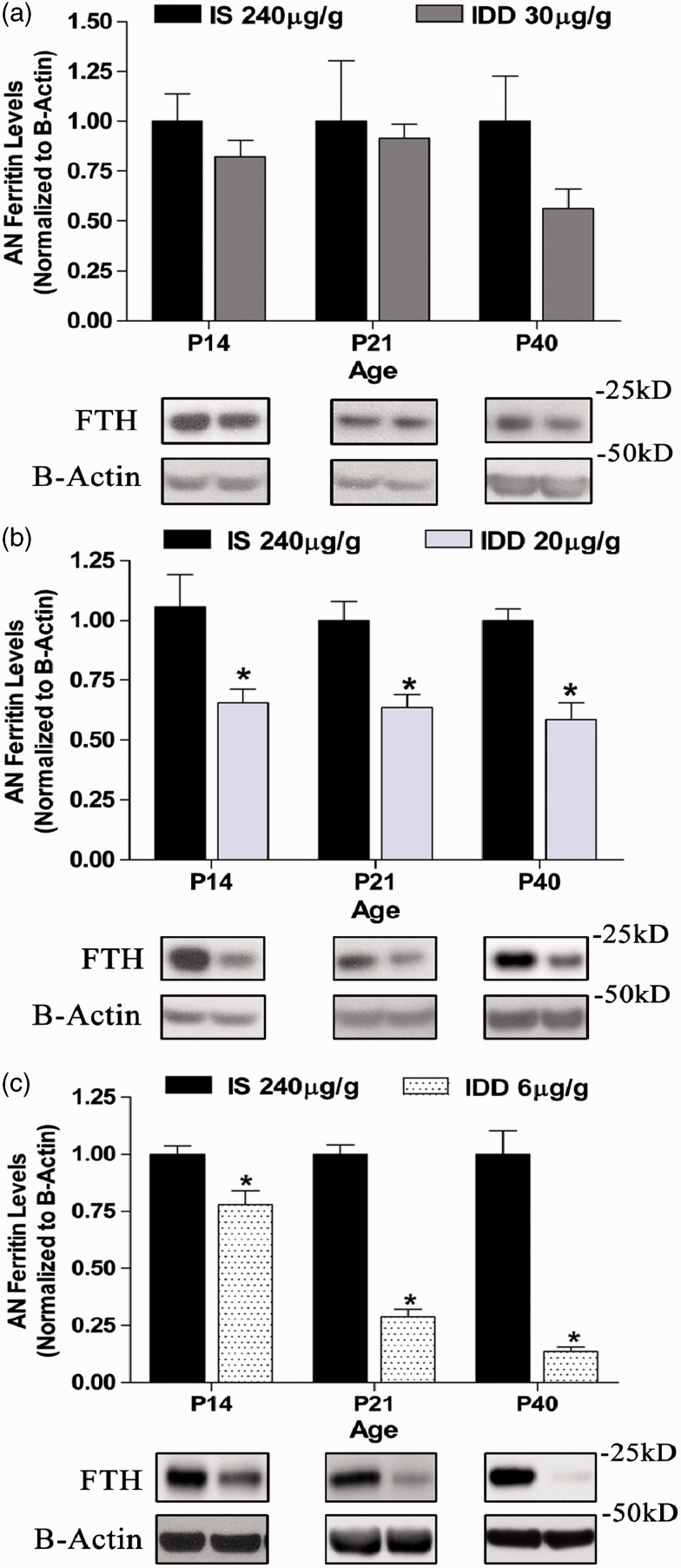
Iron store mobilization in AN tissue of offspring fed an IDD compared with the IS group. Ferritin protein expression in the IDD-30 group (a), IDD-20 group (b), and IDD-6 group (c). Data are mean ± *SEM*, *n* = 4 offspring per age and diet group. Representative Western blot bands of IS tissue (left band) and deficient tissue (right band) are shown below each age for each respective diet. Data are represented as a ratio to age-matched IS controls. *p < .05 versus age-matched IS control. IS = iron sufficient; IDD = iron-deficient diet; FTH = anti-ferritin heavy chain.

To determine whether the easy to obtain and generally used HCT and HGB provide a useful correlate for CNS tissue iron stores, particularly with respect to ABR measurements, we quantified tissue ferritin levels in the ANs of the offspring in each dietary group. As shown in Figure 2(a), tissue ferritin levels in the ANs were not different from control levels at all ages in the 30 µg Fe/g IDD group, despite the transient decrease in HCT and HGB shown in Figure 1(b) and (c). In contrast, the 20 µg Fe/g IDD significantly decreased ferritin tissue levels compared with the IS group (diet: *p* < .05; Figure 2(b)) at all ages. Interestingly, at P40 tissue, iron levels in the AN were still decreased compared with controls, while hematological parameters recovered at this time point. In the IDD-6 µg Fe/g group, ferritin protein expression was reduced in response to both diet (*p* < .001) and age (*p* < .001). There was also a significant diet by age interaction (*p* < .001), and the AN displayed a nearly 90% loss of ferritin protein expression by P40 (Figure 2(c)).

### Associations Between Tissue Ferritin Levels of the AN and Associated ABR Latencies

**Figure 3.**
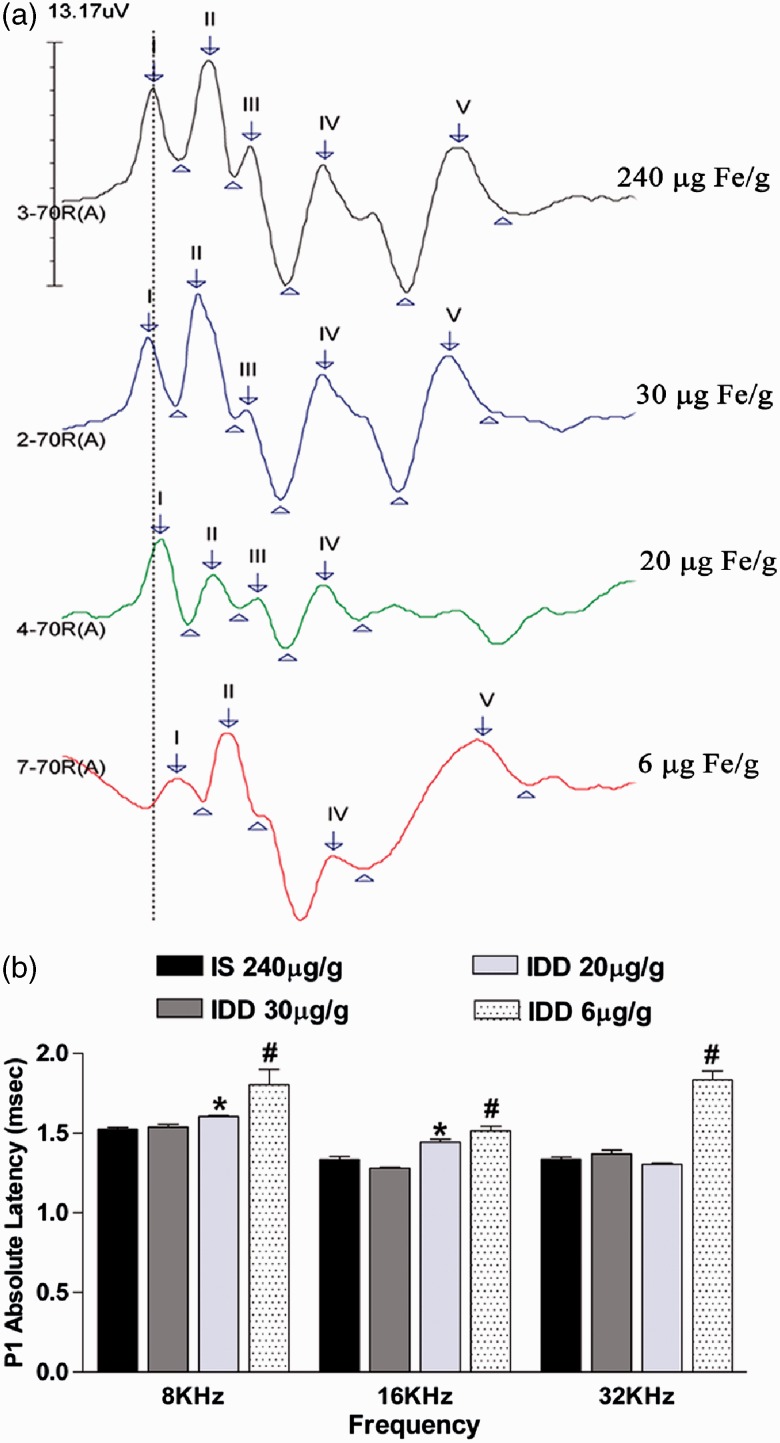
Peak 1 neuronal conduction velocity by ABR analysis in P40 offspring fed variable IDDs. (a) Representative aligned ABR potentials evoked at 16 kHz stimulus frequency and 70 dB intensity level. The dotted line demarcates the relative latency of Peak 1 (P1) in IS controls compared with each IDD group. (b) Quantified latencies from all three test frequencies in each diet group. Data are mean ± *SEM*, *n* = IS: 11, IDD-30: 9, IDD-20: 13, IDD-6: 5 rats per group. *p < .05 versus frequency-matched IS controls, ^#^*p* < .05 versus all diet groups within that frequency. IS = iron sufficient; IDD = iron-deficient diet.

To test the hypothesis that AN tissue iron depletion is a prerequisite for ABR impairments, we performed ABR analyses on P40 offspring of each diet. This age was chosen as our previous rodent models of moderate ID showed a significant decrease in neuronal conduction velocity specifically at this age (Lee et al., 2012). As shown in Figure 3, iron depletion in the AN was accompanied by significantly increased absolute latency of Peak I (PI) compared with the iron-normal AN. We observed a diet by frequency interaction (*p* < .001) indicating that the effect of diet on Peak I latency is frequency-dependent.

The 30 µg Fe/g IDD that did not reduce AN ferritin levels also was not associated with any significant latency changes in any peaks measured (Figure 3(b), Table 2).

**Table 2.** Absolute and Interpeak Latencies (ms) of P40 Offspring by ABR Analysis.

		Diet							
Stimulus (kHz)	Peak	*N*	IS–240	*N*	IDD–30	*N*	IDD–20	*N*	IDD–6
8	II	23	2.28 ± 0.02	9	2.24 ± 0.03	13	2.33 ± 0.03	5	2.57 ± 0.05^#^
	III	20	2.89 ± 0.03	6	2.89 ± 0.04	13	2.96 ± 0.03	3	3.12 ± 0.07^*^
	IV	20	3.81 ± 0.03	4	3.81 ± 0.05	13	3.91 ± 0.05	4	4.00 ± 0.09
	V	23	4.76 ± 0.07	8	5.06 ± 0.19	13	5.14 ± 0.10^*^	5	5.79 ± 0.09^#^
	II–I	22	0.69 ± 0.02	7	0.69 ± 0.03	13	0.73 ± 0.03	5	0.83 ± 0.02^*^
	V–I	21	3.20 ± 0.10	5	3.52 ± 0.21	13	3.51 ± 0.09	5	4.42 ± 0.39^#^
16	II	23	2.08 ± 0.02	8	2.07 ± 0.04	13	2.07 ± 0.03	5	2.36 ± 0.05^#^
	III	22	2.68 ± 0.03	8	2.71 ± 0.04	13	2.65 ± 0.03	4	2.89 ± 0.06^#^
	IV	23	3.69 ± 0.03	8	3.60 ± 0.06	13	3.80 ± 0.04	4	3.96 ± 0.08^#^
	V	22	5. 12 ± 0.09	7	5.27 ± 0.16	13	5.44 ± 0.04	4	5.80 ± 0.32^*^
	II–I	23	0.71 ± 0.02	8	0.67 ± 0.04	13	0.77 ± 0.02	5	0.86 ± 0.01^*^
	V–I	20	3.83 ± 0.10	4	3.84 ± 0.24	13	4.15 ± 0.13	4	4.59 ± 0.48^*^
32	II	22	2.15 ± 0.02	7	2.07 ± 0.04	13	2.13 ± 0.03	5	2.43 ± 0.05^#^
	III	23	2.78 ± 0.03	9	2.70 ± 0.05	13	2.70 ± 0.03	3	2.99 ± 0.12
	IV	23	3.78 ± 0.04	9	3.48 ± 0.08	13	3.90 ± 0.05	4	3.95 ± 0.08^#^
	V	23	5.14 ± 0.08	8	5.07 ± 0.25	13	5.52 ± 0.04^*^	5	5.64 ± 0.17^*^
	II–I	22	0.82 ± 0.02	9	0.73 ± 0.04	13	0.80 ± 0.03	5	0.95 ± 0.04^*^
	V–I	21	3.82 ± 0.09	7	3.70 ± 0.25	13	4.18 ± 0.03	5	4.39 ± 0.46

*Note.* Results are given as mean ± *SEM*. *N* represents number of peaks analyzed per group. IS = iron sufficient; IDD = iron-deficient diet. Peak 1 latencies are shown in Figure 3.

*p < .05 versus frequency-matched IS-240. ^#^p < .05 versus all frequency-matched diet groups.

Offspring in the 20 µg Fe/g IDD group displayed significant latency defects in a frequency specific manner. Specifically, PI latency was increased by 5% to 10% at 8 kHz and 16 kHz compared with both the IS group and the 30 µg Fe/g IDD group. In this group, the latency defects were restricted to Peak I, except for Peak V at 8 and 32 kHz (which were slightly [7–8%] increased) but did not result in increased PV to PI interpeak latencies (Table 2) because of the proportional increase in PI latency (Figure 3(b)).

Offspring in the 6 µg Fe/g diet group displayed significantly increased PI latency by 15% to 27% at all three stimulus frequencies compared with all other groups (*p* < .05) (Figure 3(b)). In addition to the anemia induced by the 6 µg Fe/g IDD, the absolute latencies of Peaks II to V were significantly increased by 18% to 36% depending on the peak and stimulus frequency. The 6 µg Fe/g IDD also significantly increased the interpeak latency of PII to I, indicating some disruption in the myelination status of the AN, as well as increased PV to I latencies indicating an overall defect in brainstem maturation (Table 2).

## Discussion

Although ID is a persistent public health problem and its impact on the developing CNS has been acknowledged, the lack of insight into the time period during which the fetus was exposed to ID makes it challenging to predict or diagnose the severity of CNS maturation defects (Idjradinata and Pollitt, 1993; Lozoff and Georgieff, 2006; Lee et al., 2012; Pisansky et al., 2013). Maternal or cord blood hematological parameters give an indication of the acute status of iron availability in blood but are not useful to determine the time of onset of ID or the CNS tissue iron levels. The analysis of the ABR profile has been used as a surrogate functional readout that is intended to report the maturation state of the brain; however, conflicting results in the context of ID have raised questions as to the strength of influence that ID imparts on the ABR response. A partial answer to this question is provided by our previous study (Mihaila et al., 2011), where we show in rats that if exposure to ID occurs during the later stages of pregnancy, the ABR remains normal in the offspring, even if the offspring is highly anemic and displays a low birth weight. In contrast, the ABR was significantly affected if the ID occurred at the beginning of pregnancy (Mihaila et al., 2011). As that study was designed to induce anemia in all offspring, it was impossible to clearly separate the impact of iron depletion from the impact of acute anemia, a question we approached in our previous study by Lee et al (2012). There we showed in a different rat strain that lifetime exposure to marginal ID was associated with morphological and functional defects of the AN and results in impaired ABR responses specifically in the absolute latency of Peak I. The limitation of this study was, however, that we only induced a very mild non anemic ID in the offspring.

As our two previous studies and additional published reports from others are not directly comparable due to different rat strains and diets, the goal of the present study was hence to determine whether the ABR could be utilized to detect the threshold of tissue ID in offspring exposed to lifetime ID ranging from mild to severe. This study revealed several novel findings: (a) ABR latency is impaired by mild ID that is initiated early in pregnancy and sustained during development even at levels that never reach a state of clinical ID anemia in the offspring. (b) The severity of biochemical responses to progressive iron restriction does not appear to follow a linear relationship and may indicate a dietary threshold for induction of anemia. (c) The profile of ABR latency defects resulting from moderate AN tissue ID is localized primarily to Peak I, in contrast to the broad multipeak latency defects generated by acute anemia occurring during this window of vulnerability. Taken together, these data might be relevant to humans in that they suggest that ABR latency defects in infants may indicate exposure to maternal ID that is initiated during the early stages of gestation and sustained during development. Perhaps even more important, the data also indicate that the absence of anemia in infants might not predict the absence of ID-induced maturation defects.

A novel aspect of our study is the systematic analysis of the biochemical progression of ID in an ABR relevant tissue. As the AN is too small to conduct mass spectroscopy for elementary Fe content, we used tissue ferritin as a surrogate marker for iron homeostasis. Several studies support that ferritin levels are a sensitive and specific marker of CNS iron homeostasis, and reliably decrease to facilitate the release of available cellular Fe stores (Han et al., 2000; Tran et al., 2002). The side by side analysis of hematological parameters and AN tissue iron status revealed some unexpected results. We were surprised by the disconnection between HCT, HGB, and tissue ferritin levels in the 30 µg Fe/g IDD; during the typical progression of ID toward persistent anemia, tissue ferritin levels are usually depleted prior to any observed changes in HCT and HGB. This disconnect might suggest that these slight hematological changes were not related to decreased iron availability but were a result of a slight delay in blood volume expansion to accompany the significant growth spurts that occur in weanling rats during this time (Eisen, 1976). This possibility is consistent with the observation that the decreases in HCT and HGB were mild and transient with a complete recovery by P40 without any supplementation and that no statistically significant differences were observed in postnatal growth between the two midlevel ID and the IS diet group.

The 20 µg Fe/g iron diet had an identical hematological profile as the 30 µg Fe/g but produced significantly lower ferritin levels in the AN. This might suggest that the threshold for maintaining iron saturation of the AN lies below 30 ppm dietary Fe and represents a tissue specific iron requirement. We have previously shown that ID affects the CNS in a highly region specific manner with some regions displaying far less sensitivity to iron depletion than others (Greminger et al., 2014). Interestingly, the ferritin levels remained constant over time despite a continuous 20 µg Fe/g iron diet, suggesting that the AN ferritin levels are relatively resilient to iron depletion.

In light of the ferritin profile in the 6 µg Fe/g, it seems that the threshold between AN tissue ID and anemia lies between 20 and 6 µg Fe/g dietary iron. It needs to be stressed that results of the 6 µg Fe/g diet may actually underestimate the effect of anemia on the ABR in the Long-Evans rat strain, as approximately 50% of the offspring in this diet group did not survive to the testing age, removing the most severely affected rats from our analysis and likely attenuating the latency defect. A limitation of our study here is that we focused on the most frequently used HCT and HGB values to relate the peripheral state of ID to the iron status in the AN. We can thus not exclude the possibility that serum ferritin or transferrin levels would have provided a better peripheral surrogate measurement for AN maturation defects than HCT and HGB. While outside the scope of this study, it will be important in future studies to examine suitable surrogate measurement that more reliably predict CNS tissue iron levels.

Our result on the impact of various levels of ID on the ABR response also raises the question of the underlying “anatomical locus” of pathology that results in impaired ABR responses. While the ABR profiles allow exclusion of cochlea defects on the level of haircell function, we cannot deduct from our study which anatomical defect contributed to the Peak I latency impairments in the borderline versus severe ID diet. On the basis of our previous findings (Mihaila et al., 2011; Lee et al., 2012), we suggest that the underlying pathology in the borderline diet is due to a defect in axon maturation with no impairment in myelination. In contrast, the underlying pathology for Peak I latency in the more severe anemia inducing diet might contain an additional component of defective myelination.

In summary, our study suggests that lifetime exposure to ID results in impaired ABR. The severity of the ABR impairment seems to reflect the severity of the ID, which in our study ranged from borderline ID to systemic anemia. The impact of even mild ID on the ABR is of particular clinical concern as mild ID without reaching stages of anemia might go undetected. As the CNS tissue ID in infants is difficult to deduct and commonly used hematological parameters do not reliably reflect CNS iron levels, we suggest that ABR analysis might be a useful tool to retrospectively determine whether an infant might have been exposed to ID during gestation even if no signs of maternal of infant anemia can be established.

## Summary

Iron deficiency significantly impacts neuronal function in the absence of persistent anemia. We show that auditory brainstem response analysis identified the threshold of iron deficiency in the rat central nervous system and support the need for evaluation of its diagnostic potential in at-risk children.

## Supplementary Material

Supplementary material
