# Supplementary material for: Identifying the Threshold of Iron Deficiency in the Central Nervous System of the Rat by the Auditory Brainstem Response
Source: ASN Neuro. 2015 Feb 19;7(1):1759091415569911. doi: 10.1177/1759091415569911 (PMC4366421; doi:10.1177/1759091415569911)
Supplement: Supplementary material [file Supplemental_Material_Table_S1.pdf]

Online Supplemental Material

Supplemental Table S1. Composition of Harlan Teklad IS (TD.05656) and ID (TD.80396) purified diets.

	Diet	
	**IS**	**IDD**
**Ingredient**	***g/kg***	
Casein, low Cu & Fe	200.0	200.0
DL-Methionine	3.0	3.0
Sucrose	548.6	549.9
Corn Starch	150.0	150.0
Corn Oil	50.0	50.0
Mineral Mix[2], Fe deficient (81062)	35.0	35.0
Vitamin Mix[3], AIN-76A (40077)	10.0	10.0
Ferric Citrate[1]	1.4	-
Choline Bitartate	2.0	2.0
Ethoxyquin	0.01	0.01

[1] The IDD-30, IDD-20, and IDD-6 diets contained 0.154, 0.102 and 0.0 g/kg added Ferric Citrate respectively.

[2] Vitamin mix contained the following (g/kg mix): thiamin HCl, 0.60; riboflavin, 0.60; pyridoxine HCl, 0.70; niacin, 3.0; calcium pantothenate, 1.60; folic acid, 0.20; biotin, 0.02; vitamin B12, 1.0; dry vitamin A palmitate (500,000 U/g), 0.80; dry vitamin E acetate (500U/g), 10.0; vitamin D3 trituration (400,00U/g), 0.30; menadione sodium bisulfite, 0.20; sucrose, 981.1.

[3] Mineral mix contained the following (g/kg mix): $CaHPO_4$, 500.0; NaCl, 74.0; $C_6H_7K_3O_8$, 220.0; K_2SO_4, 52.0, MgO, 24.0; $MnCO_3$, 3.5; $ZnCO_3$, 1.6; $CuCO_3$, 0.3; KIO_3, 0.01; $Na_2SeO_3 \cdot$ 5H2O, 0.01; $KCrS_2O_8$, 0.6; sucrose 124.0.
